# Supplementary material for: ALS-Associated SOD1(G93A) Decreases SERCA Pump Levels and Increases Store-Operated Ca2+ Entry in Primary Spinal Cord Astrocytes from a Transgenic Mouse Model
Source: Int J Mol Sci. 2019 Oct 17;20(20):5151. doi: 10.3390/ijms20205151 (PMC6829245; doi:10.3390/ijms20205151)
Supplement: Supplementary file 1 [file ijms-20-05151-s001.pdf]

ALS-Associated SOD1(G93A) Decreases SERCA Pump Levels and Increases Store-Operated Ca^{2+} Entry in Primary Spinal Cord Astrocytes from a Transgenic Mouse Model

Rosa Pia Norante [1], Caterina Peggion [1], Daniela Rossi [2], Francesca Martorana [2], Agnese De Mario [1], Annamaria Lia [1], Maria Lina Massimino [3], Alessandro Bertoli [1,3,4*]

[1] Department of Biomedical Sciences, University of Padova, Padova, 35131, Italy

[2] Laboratory for Research on Neurodegenerative Disorders, Istituti Clinici Scientifici Maugeri SpA SB – IRCCS, Pavia, 27100, Italy

[3] CNR – Neuroscience Institute, University of Padova, Padova, 35131, Italy

[4] Padova Neuroscience Center, University of Padova, Padova, 35131, Italy

* Correspondence: alessandro.bertoli@unipd.it; Tel.: 39-049-827-6150

SUPPLEMENTARY MATERIAL

Supplementary Materials and Methods

Immunocytochemistry

For immunocytochemical assays, astrocytes were plated onto glass coverslip at a density of 80,000 cells/well in 24-well plates. After 96 h from plating, cells were firstly washed in ice-cold phosphate-buffered saline (PBS), and then fixed (20 min, RT) in paraformaldehyde [4% (w/v) in PBS]. After washing in PBS, cells were permeabilized (1 h, RT) in PBS containing Triton X-100 [0.02% (w/v)] and then incubated (overnight, 4 °C) with a rabbit anti-GFAP polyclonal (p) antibody (Ab) (1:500, Dako, cat. n. Z0334).

After extensive washings in PBS, cells were incubated (1 h, 37 °C) with a TRITC-conjugated anti-rabbit IgG secondary Ab (1:100, Dako, cat. n. R0156). Cell nuclei were counter-stained with Hoechst 33342 (5 μg/ml, Sigma), and coverslips were finally washed in PBS, and mounted in montage solution [Mowiol 40-88, 8% (w/v), Sigma] in a glycerol:PBS [1:3 (v/v) solution]. Cells were observed with an inverted fluorescence microscope (Leica CTR6000) equipped with a computer-assisted charge-coupled camera (Orca Flash 4.0, Hamamatsu), which allowed the acquisition and analysis of digital images.

Quantitative Real-time RT-PCR

Total RNA was extracted from mouse astrocytic cultures using RNeasy® Mini kit according to manufacturer's guidelines (Qiagen). 0.5-1 μg of extracted total RNA was reverse transcribed using IScript™ cDNA Synthesis kit according to manufacturer's instructions (Bio-Rad Laboratories). The resulting cDNAs (2 ng) were analyzed by quantitative PCR using the SsoFast EvaGreen Supermix on a CFX96 Real-Time PCR Detection System (Bio-Rad Laboratories). Relative expression was determined by the $2^{-◎◎ct}$ method and normalized to

hypoxanthine-guanine phosphoribosyl transferase gene expression. Primer sequences for gene amplification are available upon request.

Western Blot Analysis

For the analysis of protein glutathionylation, cells were lysed in a solution containing glycerol 10% (w/v), SDS 2% (w/v), Tris-HCl 62.5 mM (pH 6.8), a protease inhibitor cocktail (Roche) and iodoacetamide 30 mM, for 1h at RT in the dark. Proteins were separated on 4–12% Bis-Tris gels (Bio-Rad) under native nonreducing conditions. For all other Western blot (WB) procedures, see Materials and Methods (paragraph 4.6) in the main text.

<u>Antibodies</u> used in supplementary Figure S6 were a mouse monoclonal Ab recognizing all plasma membrane (PM) Ca^{2+} ATPase (PMCA) isoforms (1:2000; Sigma, Saint Louis, MO, USA, cat. n. P6363), and a rabbit pAb recognizing all PM Na^{+}/Ca^{2+} exchanger (NCX) isoforms (H300, Santa Cruz Biotechnology, Dallas, TX, USA, cat. n. sc-515768). In supplementary Figure S12 a mouse anti-GSH monoclonal antibody (1:500, Virogen, Watertown, MA, USA, cat. n. 101-A) was used.

Supplementary Figure S1

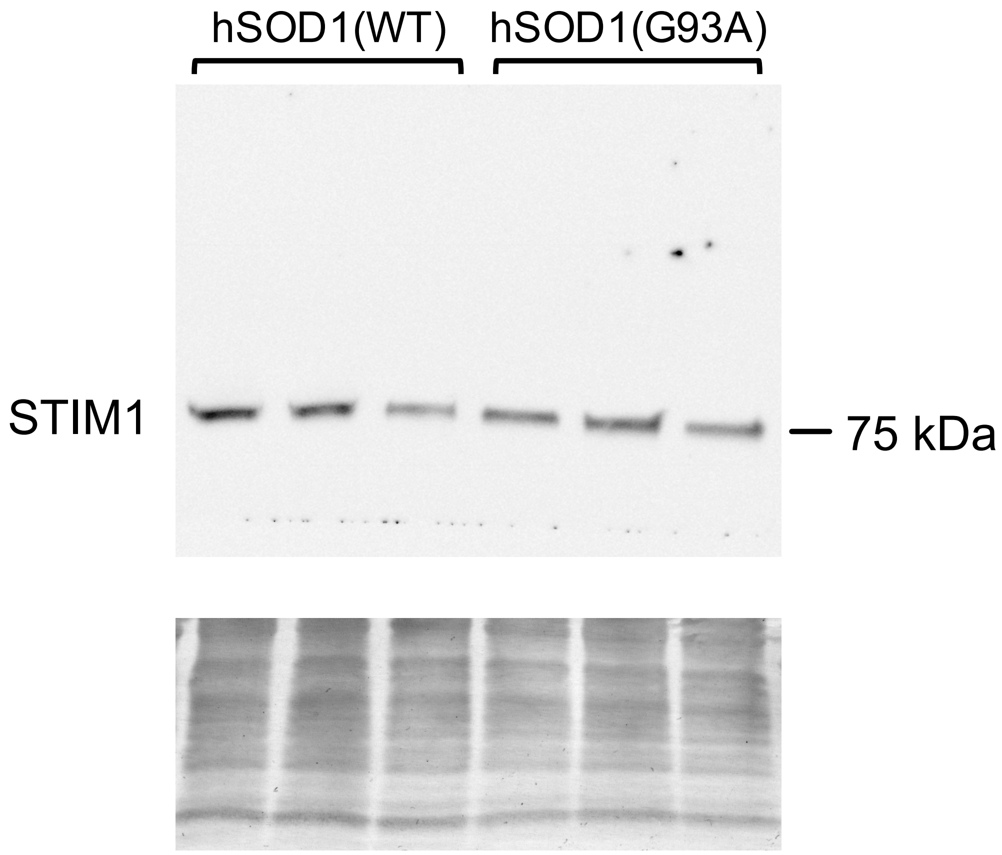

Figure S1. Full-size image of the STIM1 WB (upper panel) and the Coomassie stained PVDF membrane (lower panel) reported in Fig 2A.

Supplementary Figure S2

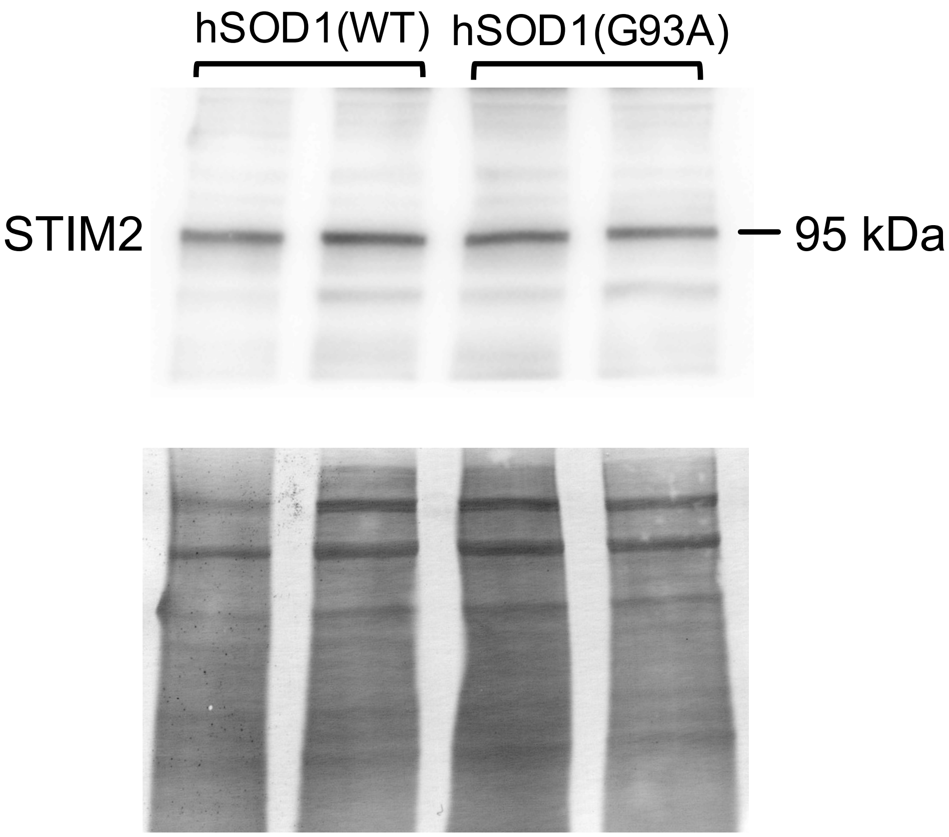

Figure S2. Full-size image of the STIM2 WB (upper panel) and the Coomassie stained PVDF membrane (lower panel) reported in Fig 2B.

Supplementary Figure S3

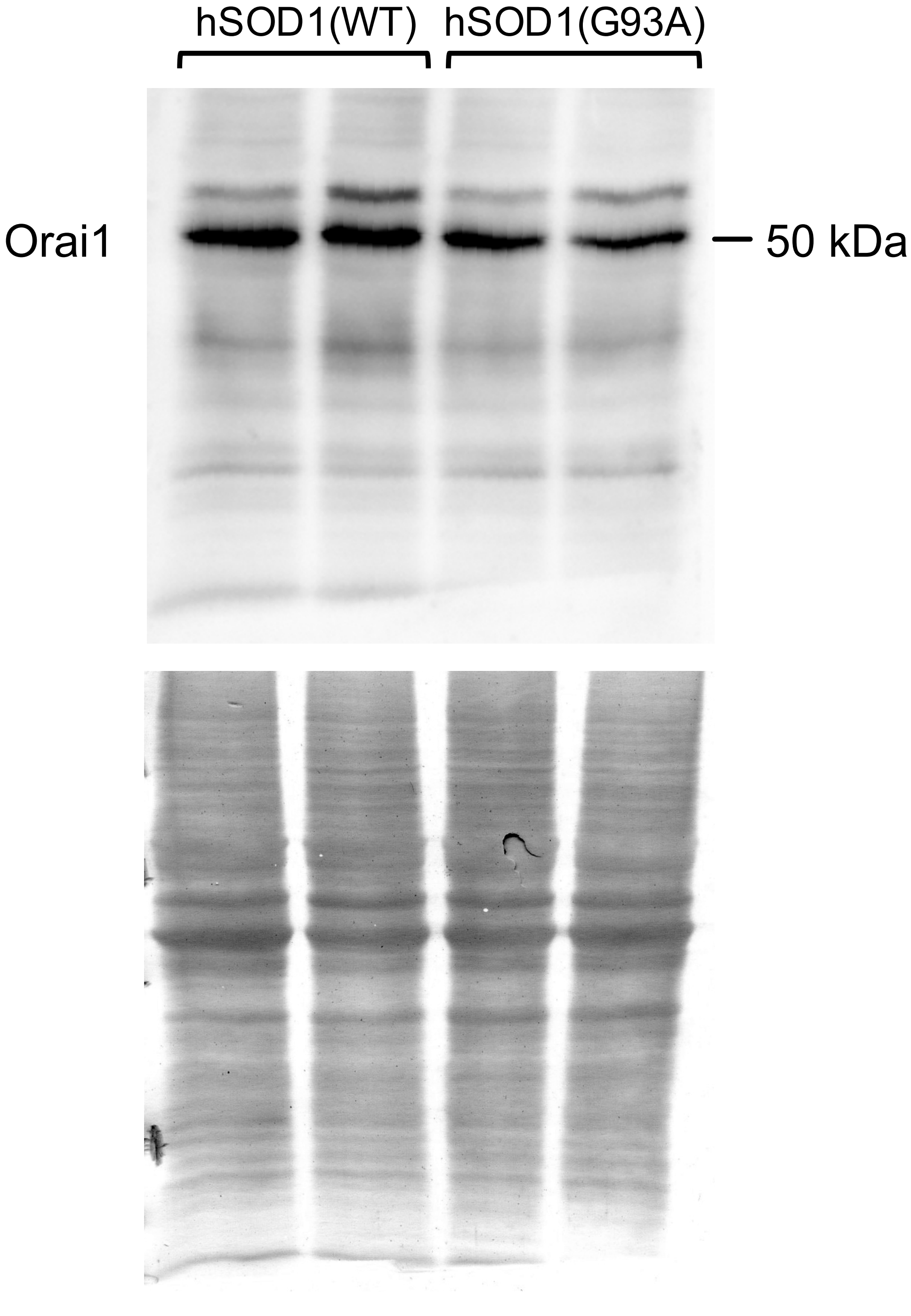

Figure S3. Full-size image of the Orai1 WB (upper panel) and the Coomassie stained PVDF membrane (lower panel) reported in Fig 2C.

Supplementary Figure S4

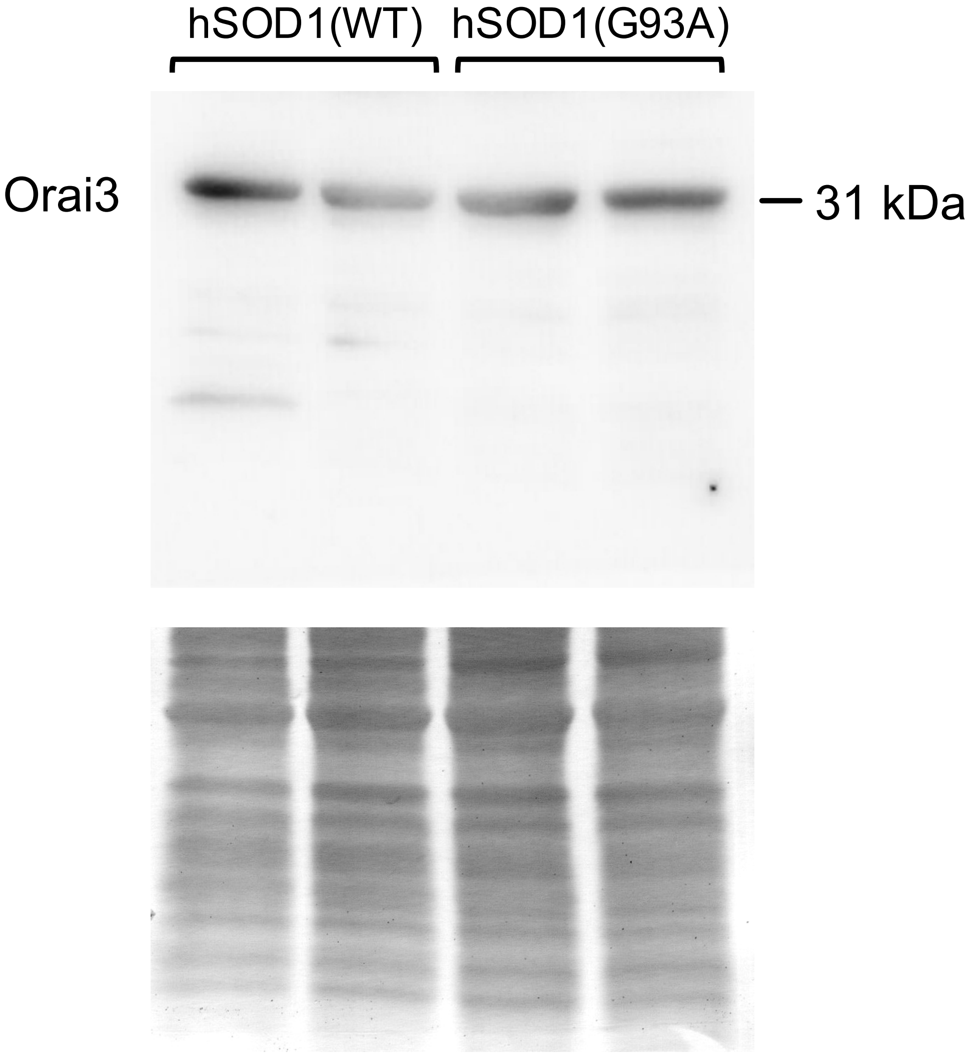

Figure S4. Full-size image of the Orai3 WB (upper panel) and the Coomassie stained PVDF membrane (lower panel) reported in Fig 2D.

Supplementary Figure S5

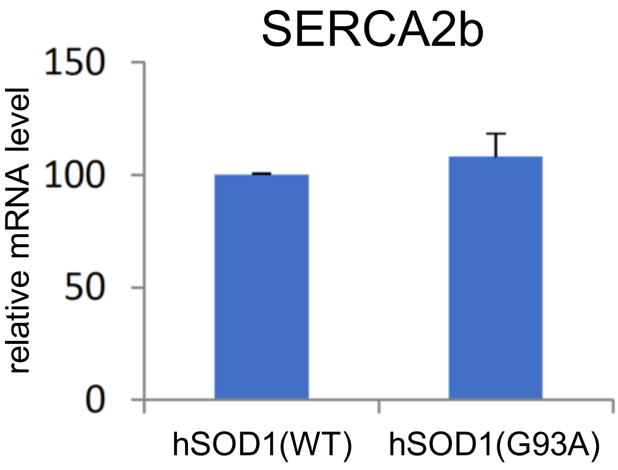

Figure S5. mRNA levels of the SERCA2b isoform are comparable in fALS and control primary astrocytes. Total cDNA, obtained by reverse transcription of mRNA extract from hSOD1(WT) or hSOD1(G93A) primary astrocytic cultures, was subjected to quantitative real-time PCR using specific primers for the SERCA2b isoform (the alternative splicing variant of SERCA2 most abundant in the nervous system). Hypoxanthine-guanine phosphoribosyl transferase was used as housekeeping gene, and data are reported in the bar diagram as percentage of the hSOD1(WT) value. No significant difference is appreciable between the two hSOD1 genotypes. n = 9 independent cultures for each mouse strain.

Supplementary Figure S6

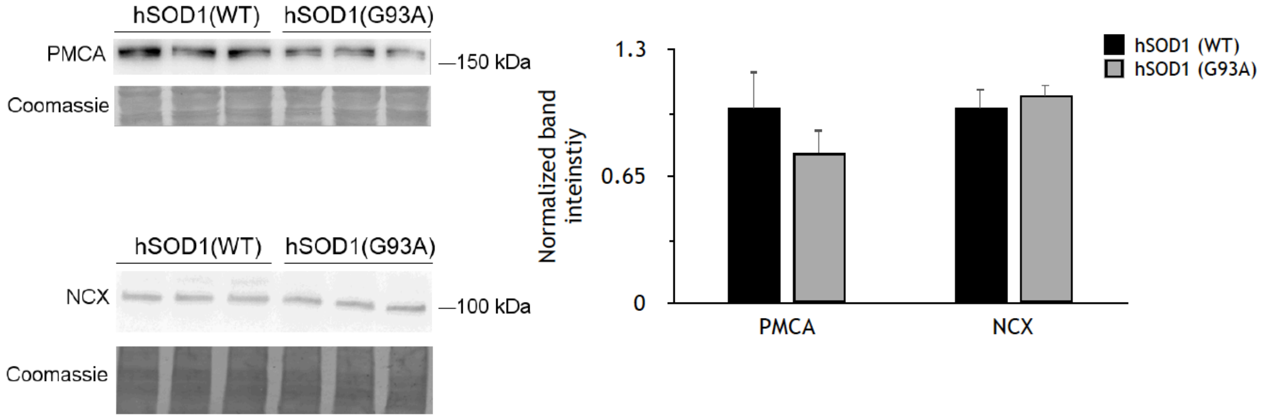

Figure S6. hSOD1(WT) and hSOD1(G93A) astrocytes express comparable levels of PMCA and NCX. Protein extracts from primary astrocyte cultures were analyzed by WB for the expression of the two PM Ca^{2+}-extruding proteins PMCA and NCX. In the left panels, the upper figures report a representative WB for each of the proteins of interest (run in triplicate), and the lower figures show the corresponding Coomassie blue-stained membrane. Molecular weights are reported on the right of each WB panel. The bar diagram in the right panel shows the densitometric analysis of immunoreactive bands for the different proteins normalized to the optical density of the corresponding Coomassie blue-stained lane and reported as fraction of the SOD1(WT) values. No significant difference exists for either of the two proteins between hSOD1(WT) and hSOD1(G93A) astrocytes. n = 6 independent cultures for each hSOD1 genotype. Full-size images of WBs are reported in supplementary Figs. S17 and S18.

Supplementary Figure S7

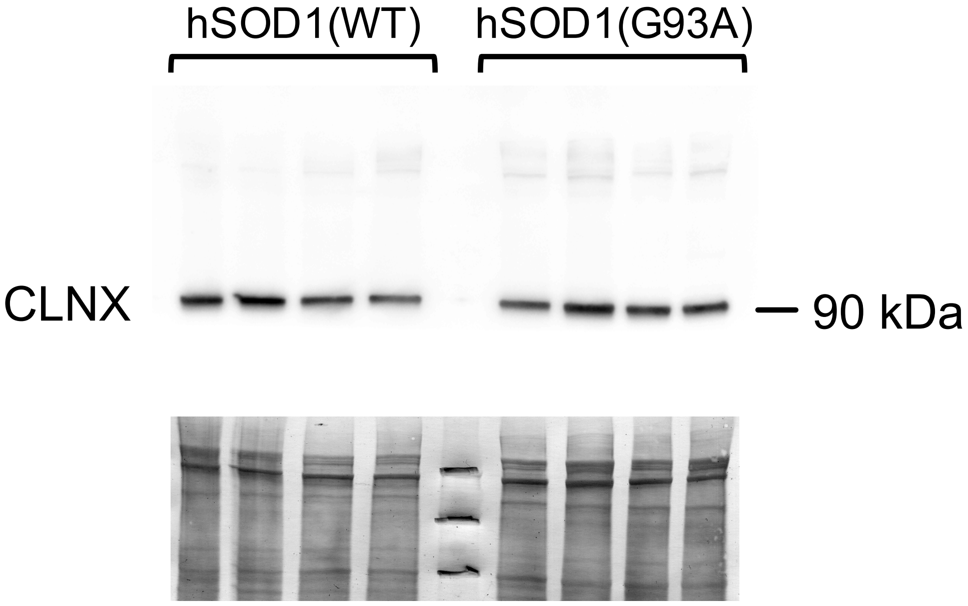

Figure S7. Full-size image of the CLNX WB (upper panel) and the Coomassie stained PVDF membrane (lower panel) reported in Fig 4A.

Supplementary Figure S8

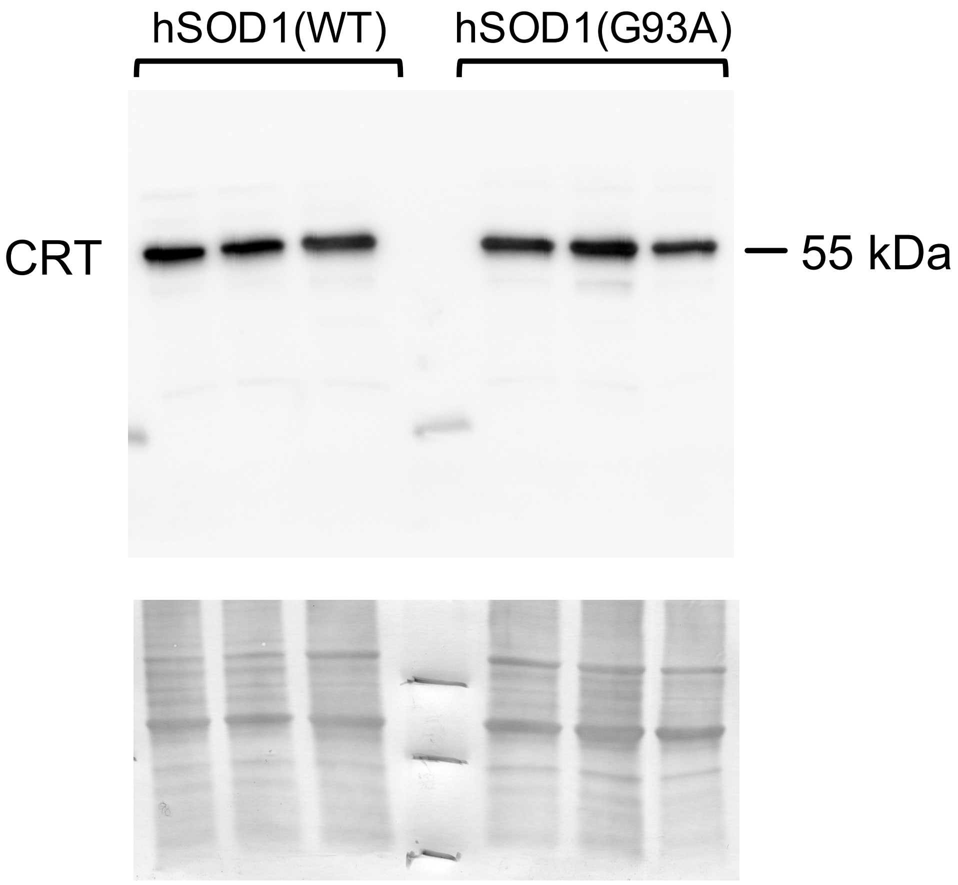

Figure S8. Full-size image of the CRT WB (upper panel) and the Coomassie stained PVDF membrane (lower panel) reported in Fig 4B.

Supplementary Figure S9

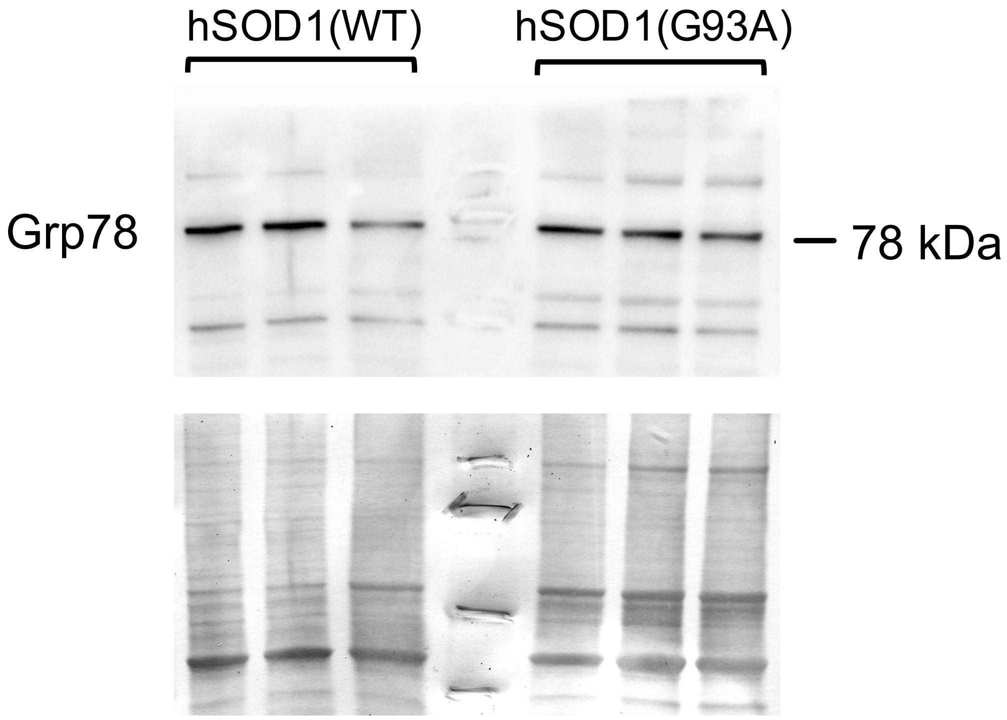

Figure S9. Full-size image of the Grp78 WB (upper panel) and the Coomassie stained PVDF membrane (lower panel) reported in Fig 4C.

Supplementary Figure S10

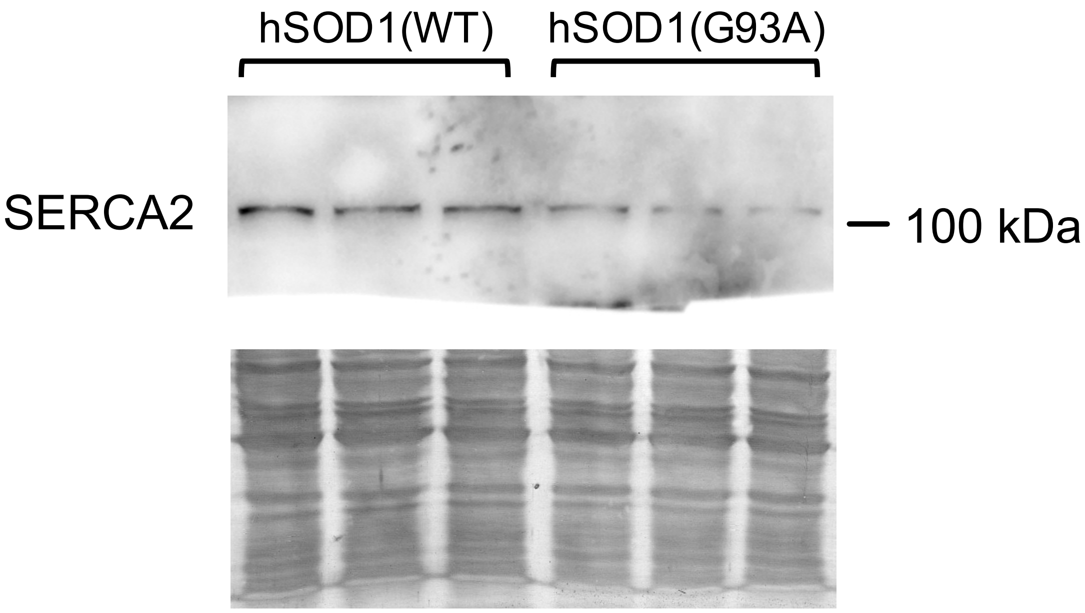

Figure S10. Full-size image of the SERCA2 WB (upper panel) and the Coomassie stained PVDF membrane (lower panel) reported in Fig 4D.

Supplementary Figure S11

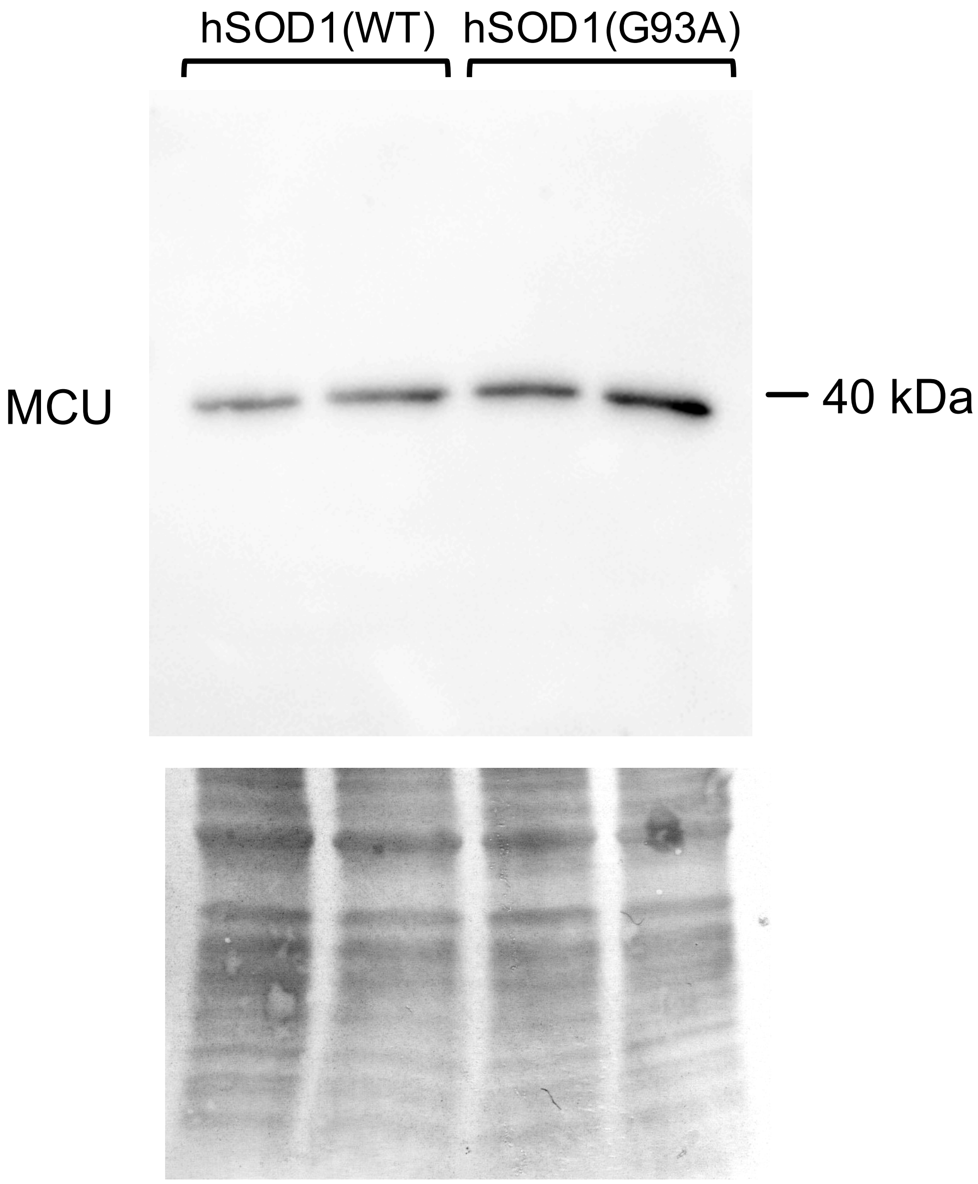

Figure S11. Full-size image of the MCU WB (upper panel) and the Coomassie stained PVDF membrane (lower panel) reported in Fig 6B.

Supplementary Figure S12

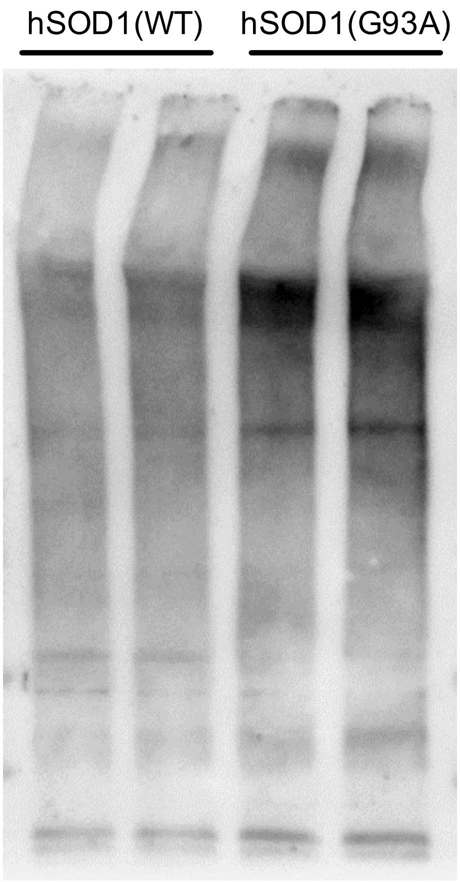

Figure S12. hSOD1(G93A) astrocytes have higher levels of total protein glutathionylation compared to the hSOD1(WT) counterpart. Protein extracts from two different primary cultures of astrocytes for each hSOD1 transgene were analyzed by WB for the presence of glutathionylated proteins by a mouse anti-GSH monoclonal antibody. The increased immunosignal observed in two independent cultures of SOD1(G93A) astrocytes compared to the hSOD1(WT) counterparts is suggestive of increased protein glutathionylation in cultured astrocytes expressing the ALS-associated hSOD1 mutant.

Supplementary Figure S13

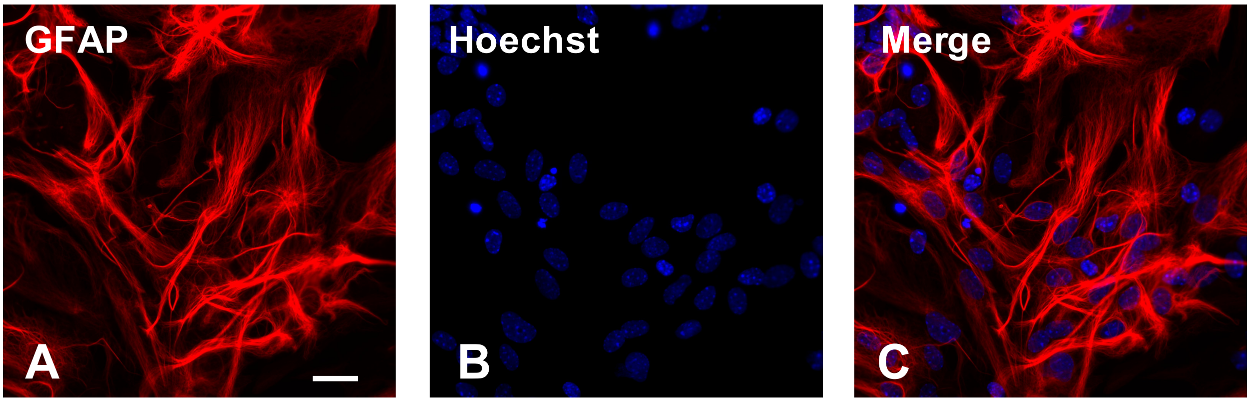

Figure S13. Used primary cultures were almost pure in astrocytes. 96 h after plating, primary astrocytic cultures from the spinal cord of newborn hSOD1(G93A) mice were fixed, permeabilized and fluorescence immunostained with an antibody to the astrocytic marker GFAP (panel A, red signal), then counterstained with the dye Hoechst 33342 marking all nuclei (panel B, blue signal), and observed with a fluorescence microscope. Merge of the two images (panel C) shows that the great majority of cells in the culture are GFAP-positive astrocytes. Scale bar, 60 μm.

Quantitative assessment of GFAP-positive cells (performed on 18 random fields from 3 different primary cultures) resulted in a 95.2% ± 0.6% (mean ± SEM) percentage of astrocytes with respect to total cells. Analysis of primary cultures from hSOD1(WT) mice provided comparable results.

Supplementary Figure S14

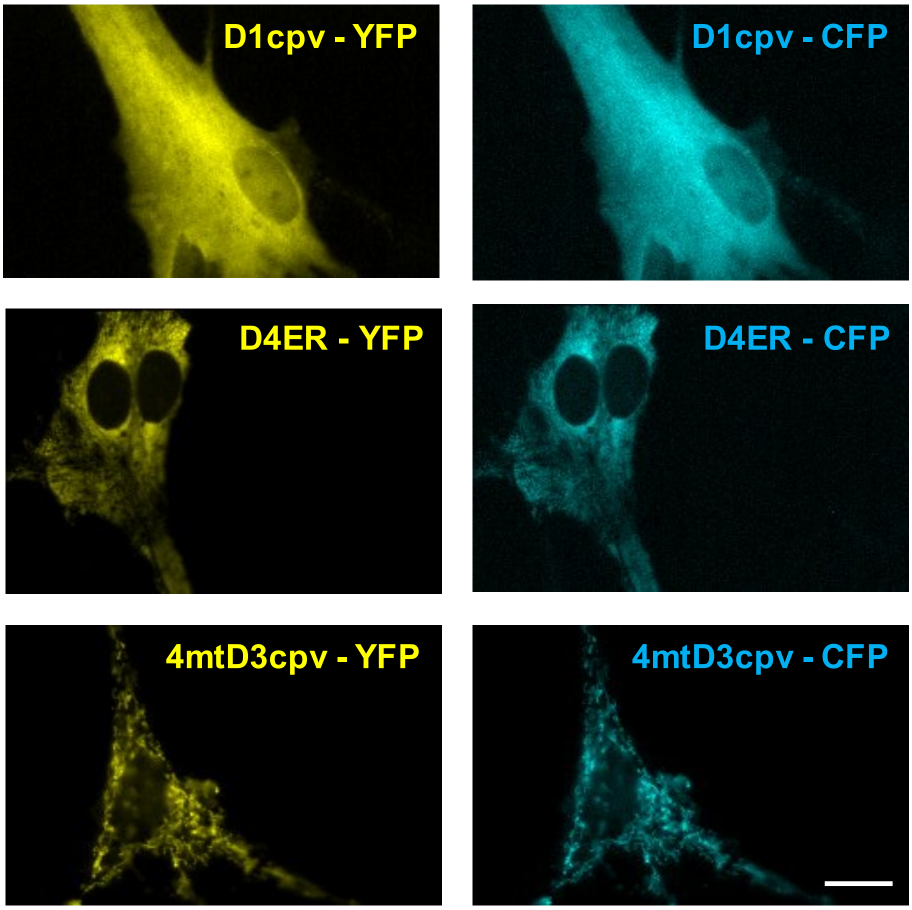

Figure S14. Representative fluorescence micrographs of astrocytes expressing the cameleon probes D1cpv, D4ER or 4mtD3cpv targeted to the cytosol, the ER lumen or the mitochondrial matrix, respectively, under the conditions used for basal Ca^{2+} measurements reported in Figure 3 (panels A and C) and Figure 6 (panel A). Both signals of the FRET-acceptor (yellow fluorescent protein, YFP) and FRET-donor (cyan fluorescent protein, CFP), collected at 535 nm and 480 nm emission wavelengths, respectively, are reported. Scale bar, 20 μm.

Supplementary Figure S15

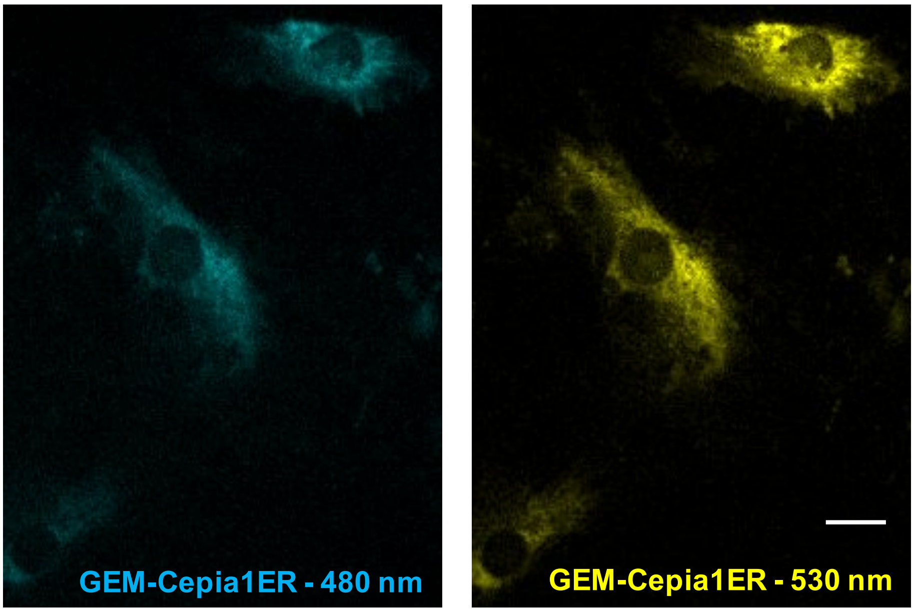

Figure S15. Representative fluorescence micrographs of astrocytes expressing the GEM-Cepia1ER genetically-encoded Ca^{2+}-indicator targeted to the ER lumen, under the conditions used for basal Ca^{2+} measurements reported in Figure 3D. Both signals at 480 nm (Ca^{2+}-bound indicator, cyan signal) and 530 nm (Ca^{2+}-free indicator, yellow signal) emission wavelengths are reported. Scale bar, 20 μm.

Supplementary Figure S16

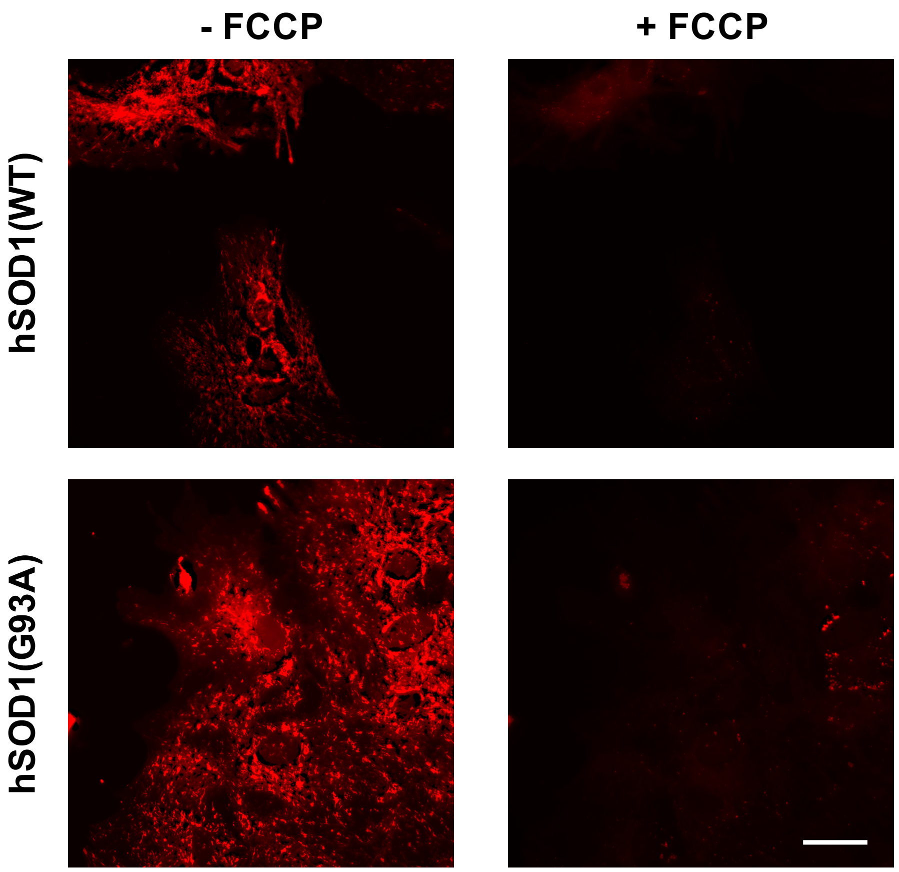

Figure S16. Representative fluorescence micrographs of hSOD1(WT)- and hSOD1(G93A)-expressing astrocytes, loaded with the cationic membrane-permeant TMRM fluorescent dye (λ_{ex}=548 nm, λ_{em}=574 nm) for the measurement of the mitochondrial membrane potential ($\Delta\psi_m$), as described in Materials and Methods, paragraph 4.4, before (-) or after (+) addition of the mitochondrial oxidative phosphorylation uncoupler FCCP (5 μM). Scale bar, 20 μm.

Supplementary Figure S17

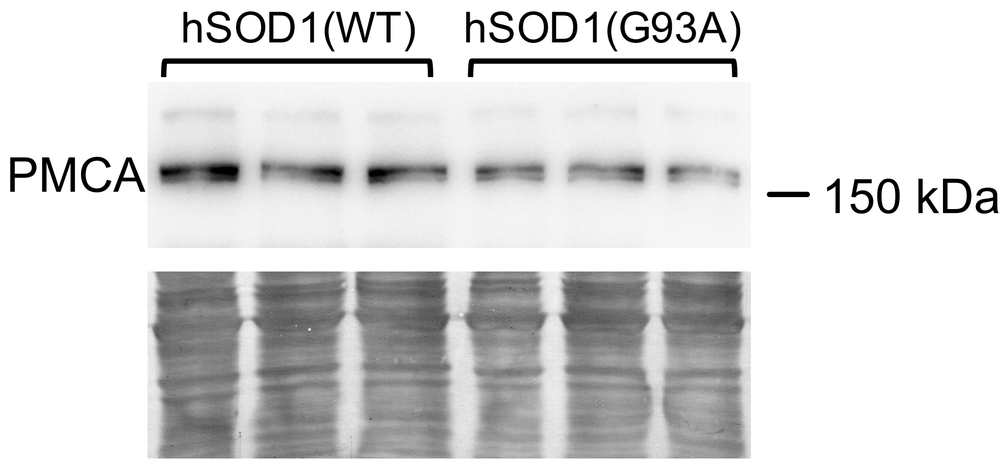

Figure S17. Full-size image of the PMCA WB (upper panel) and the Coomassie stained PVDF membrane (lower panel) reported in supplementary Fig S6.

Supplementary Figure S18

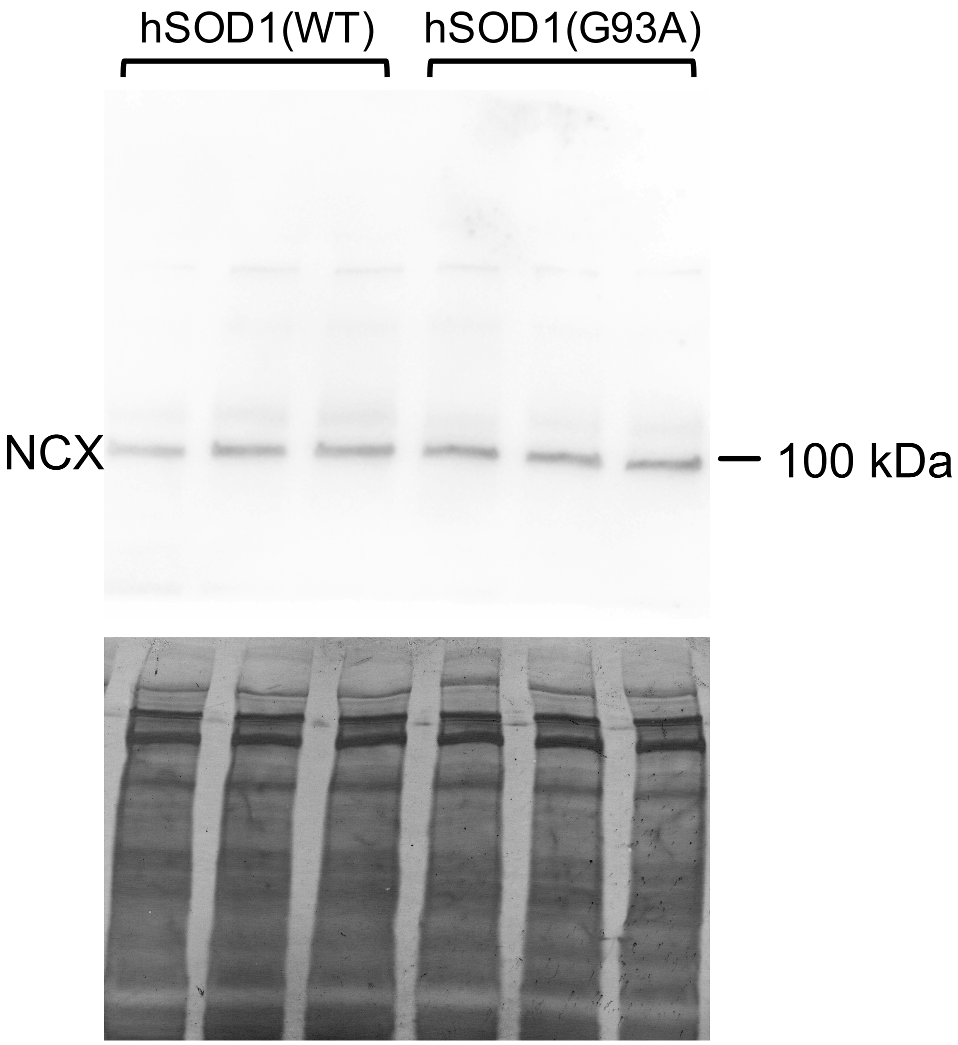

Figure S18. Full-size image of the NCX WB (upper panel) and the Coomassie stained PVDF membrane (lower panel) reported in supplementary Fig S6.
